# Prediction of Tensile Behavior of UHSFRC Considering the Flow Field in the Placing Dominated by Shear Flow

**DOI:** 10.3390/ma11020194

**Published:** 2018-01-26

**Authors:** Joon-Shik Moon, Su-Tae Kang

**Affiliations:** 1 Department of Civil Engineering, Kyungpook National University, 80 Daehakro, Bukgu, Daegu 41566, Korea; J.moon@knu.ac.kr; 2 Department of Civil Engineering, Daegu University, 201 Daegudae-ro, Jillyang, Gyeongsan 38453, Korea

**Keywords:** UHSFRC, steel fiber, fiber orientation, shear flow, tensile behavior, J0101

## Abstract

Considering the case of fabricating a UHSFRC (ultra-high strength fiber-reinforced concrete) beam with the method of one end placing and self-flowing to the other end, it was intended to simulate the variation of the fiber orientation distribution according to the flow distance and the variation of the resultant tensile behaviors. Then the validity of the simulation approach was shown by comparing the simulated results with experimental ones. A three-point bending test with a notched beam was adopted for the experiment and a finite element analysis was performed to obtain the simulated results for the bending test considering the flow-dependent tensile behavior of the UHSFRC. From the simulation for the fiber orientation distribution according to the flow distance, it could be found that the major change in the fiber orientation distribution took place within a short flow distance and most of the fibers became nearly aligned to the flow direction. After some flow distance, there was a not-so-remarkable variation in the fiber orientation distribution that could influence the tensile behavior of the composite. For this flow region, the consistent flexural test results, regardless of flow distance, demonstrate the reliability of the simulation.

## 1. Introduction

Short-fiber reinforced cementitious composite is now widely used for construction materials because it provides better mechanical properties, such as improved tensile strength, crack resistance, energy adsorption capacity, etc., compared to normal concrete without short-fiber reinforcement. Recently, several kinds of high-performance short-fiber reinforced cementitious composites have been developed [1,2,3,4,5]. Among them, ultra-high strength fiber-reinforced concrete (UHSFRC), which was developed in the early 1990s and thereafter has been actively researched, exhibits very high compressive strength of 200 MPa, more or less, and excellent durability [4,5,6,7]. Incorporation of steel fibers provides considerably improved ductility in compressive and tensile failure despite its very high strength. The direct tensile strength of UHSFRC reaches more than 10 MPa, which corresponds to roughly between a half and a third of the compressive strength of normal strength concrete, and the flexural tensile strength is more than 35 MPa [8,9,10,11,12]. Thanks to the excellent mechanical performance and durability, there have been extensive applications to various structures, such as bridges and buildings, and the continuous growth of its application is expected [13,14]. 

In general, when the fiber is mixed with concrete, the material properties of the concrete which is improved most greatly are the tensile strength and ductility. However, the improvement in tensile strength and ductility is significantly varied depending on the characteristics of fiber, such as geometry, volume fraction, tensile strength, dispersion, arrangement, etc. Therefore, the tensile strength or tensile behavior of a fiber-reinforced concrete is generally expressed as a function of some of these influencing factors [15,16,17,18,19,20,21,22,23,24]. The most common variables used for the functions are fiber volume fraction, fiber aspect ratio (length/diameter ratio), and fiber orientation. Among them, the volume fraction and the aspect ratio of the fibers are determined at the stage of mix design, while the fiber orientation is strongly dependent on the geometry of the structural member to be fabricated and the casting method at the actual construction stage.

In typical fiber-reinforced concrete containing coarse aggregate, the fiber orientation distribution changes slightly depending on the geometry of the structural member and, thus, its effect on the tensile strength has been normally considered with the introduction of the fiber orientation coefficient. However, the effect of fiber orientation on the tensile strength is slight compared with that of the fiber volume fraction and aspect ratio. However, for UHSFRC, due to its high flowability and viscosity of the matrix without the use of coarse aggregate, the fiber orientation distribution significantly varies with the flow of the fresh composite, which eventually results in very large variations in the tensile strength and behavior [12,24,25,26,27,28,29]. The effect of fiber orientation is as much as, or more than, the one of fiber volume fraction and aspect ratio for UHSFRC. 

There are two methods available to consider this serious variation of tensile performance of UHSFRC according to the fiber orientation distribution in a structural design. The first method is to apply the tensile behavior model modified by using a coefficient which can consider the uncertainty due to the effect of the fiber orientation and, thus, sufficiently ensure the safety of the designed structure [30,31,32]. The second method is to directly measure the fiber orientation distribution and the tensile performance according to the location in a trial structure, which is preliminarily fabricated in the same casting manner before casting the target structure, and then to reflect the obtained location-dependent tensile performances to the structural design [30]. However, the first method includes many uncertainties and the second method requires cost- or time-consuming work.

If the variation of the fiber orientation distribution according to the fluid flow can be predicted by considering the matrix of the composite as a fluid and the fibers as suspensions, it is possible to mitigate the uncertainty of the coefficient in the first method and also save the cost and time for the preliminary estimation in the second method. The prediction of the fiber orientation distribution makes it possible to reduce the unexpected variability of the structural performance caused by the difference in the fiber orientation distribution, leading to a reliable, as well as economic, design and construction of a UHSFRC structure. Moreover, sometimes the performance of the structure may be efficiently improved by utilizing the anisotropy of the mechanical properties of UHSFRC. 

Therefore, it is necessary to study a method to predict the fiber orientation distribution in the flow field generated in the casting process for viscous fluids such as cement mortar. As a part of the study, it was intended to predict the variation of fiber orientation distribution according to the flow distance of fresh UHSFRC with the flow field generated in the case of making a beam with a rectangular cross-section, then to predict the fiber bridging resistance and the flexural tensile performance according to the location in the beam.

## 2. Prediction Model

### 2.1. Prediction of Fiber Orientation Distribution According to the Flow Distance in Shear Flow

In 1922, Jeffery [33] developed an equation about flow-dependent rotational motion of a rigid ellipsoidal particle immersed in a homogeneous fluid based on hydrodynamics. This equation enables us to predict the fiber orientation of a steel fiber along the flow of steel fiber-reinforced cementitious composite. 

With the assumption that there is no fiber-to-fiber interaction, Jeffery’s equation for the rotational motion of a single fiber is expressed by:

(1) $${\dot{p}}_{i}=-\frac{1}{2}\omega_{ij}p_{j}+\frac{1}{2}\lambda \left({\dot{\gamma }}_{ij}p_{j}-{\dot{\gamma }}_{kl}p_{k}p_{l}p_{i}\right)$$

**Figure 1:**
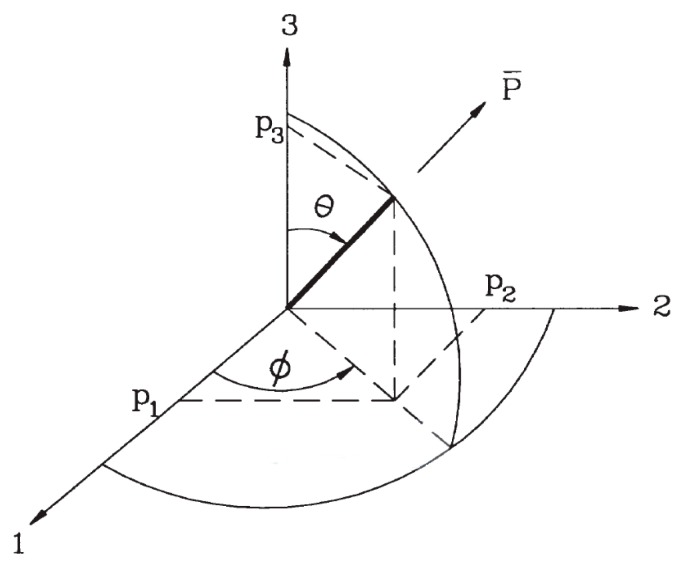
Coordinate system to express the fiber orientation.

In Equation (1), $p_{i}$ indicates the component of unit direction vector representing the orientation of a single fiber. As shown in Figure 1, for a unit direction vector with θ and ϕ in the spherical coordinate system, the Cartesian coordinate system provides the components of the vector expressed as functions of θ and ϕ.

(2) $$p_{1}=\sin\theta \cos\phi \;p_{2}=\sin\theta \sin\phi \;p_{3}=\cos\theta$$

$\omega_{ij}$ means the vorticity component and ${\dot{\gamma }}_{ij}$ represents the shear strain rate component. They are expressed by Equations (3) and (4), respectively:

(3) $$\omega_{ij}=\frac{\partial V_{j}}{\partial x_{i}}-\frac{\partial V_{i}}{\partial x_{j}}$$

(4) $${\dot{\gamma }}_{ij}=\frac{\partial V_{j}}{\partial x_{i}}+\frac{\partial V_{i}}{\partial x_{j}}$$

where $V_{i}$ is the velocity component, and λ indicates a factor relevant to the aspect ratio of fiber.

The aspect ratio of a fiber, $r_{e}$ is defined as the ratio of the length ($l_{f}$) to the diameter ($d_{f}$) of the fiber, and λ is given by the following:

(5) $$\lambda =\frac{r_{e}^{2}-1}{r_{e}^{2}+1}$$

The probability density function (p) of the fiber orientation, which means the probability in which there is a fiber having the angle θ and ϕ, is defined as:

(6) p(θ,ϕ)=ψ(θ,ϕ)sinθdθdϕ

If the fibers are assumed to be randomly oriented in three dimensions at the initial state, the orientation distribution function ψ is ψ(θ,ϕ)=1/4π. The probability density function of fiber orientation changes according to the flow of the fluid. 

In order to estimate the change of fiber orientation according to the flow, the flow field needs to be defined in advance. During molding of fresh UHSFRC, the flow field is very complex and it is strongly dependent on the form shape. However, for the case of fabricating a beam with the method of one end placing and self-flowing to the other end, the flow can be assumed as a fully-developed shear flow between two parallel plates in a steady state for the sake of simplicity.

In a simple shear flow, only the $V_{x}$ component in the flow direction is non-zero, but $V_{y}$ and $V_{z}$, corresponding to the components of the other directions, are zero in the Cartesian coordinate system. Moreover, when if the flow is fully developed, the flow velocity is not varied irrespective of the flow distance and, hence, can be described as a function of y only, so that $V_{x}=V_{x}\left(y\right)$. The velocity profile $V_{x}\left(y\right)$ is a quadratic function. Newtonian, as well as Bingham, fluids can have the velocity profile in fluid dynamics. It is well known that cementitious materials, such as mortar or concrete, can be treated as Bingham fluids in the study of fluid dynamics. Therefore, the fully-developed shear flow can be adopted for the prediction of fiber orientation distribution according to the flow of UHSFRC.

### 2.2. Prediction of Uniaxial Tensile Behavior of SFRC Considering Fiber Orientation Distribution

The uniaxial tensile behavior of steel fiber reinforced composite (SFRC) can be predicted for the stage before cracking and after cracking, respectively. The tensile behavior before a cracking occurs is estimated based on the rule of mixture. Considering the influence of the orientation distribution and length of fibers, the tensile stress of the composite is expressed by the equation:

(7) $$\sigma_{c}\left(\varepsilon_{c}\right)=\sigma_{m}\left(\varepsilon_{c}\right)V_{m}+\eta_{l}\eta_{\theta }\sigma_{f}\left(\varepsilon_{c}\right)V_{f}$$

where $\varepsilon_{c}$ is the tensile strain in the composites, and $\sigma_{f}\left(\varepsilon_{c}\right)$ and $\sigma_{m}\left(\varepsilon_{c}\right)$ represent the tensile stress at a strain $\varepsilon_{c}$ in the fiber and the matrix, respectively. $V_{m}$ and $V_{f}$ indicate the volume ratio of the matrix and the fiber, and $\eta_{l}$ and $\eta_{\theta }$ represent the coefficients related to the fiber length efficiency and the fiber orientation.

The fiber length efficiency coefficient, $\eta_{l}$ is a function of fiber length and volume fraction, fiber packing arrangement, and the mechanical properties of matrix and fiber [34,35]. The coefficient $\eta_{\theta }$ can be expressed by Equation (8) considering the probability density function of the fiber orientation, p(θ) [36]:

(8) $$\eta_{\theta }=\overset{\theta_{max}}{\underset{\theta_{min}}{\int }}p\left(\theta \right)\cos^{2}\theta \;d\theta$$

where θ indicates the angle between the axis direction of a fiber and the direction the tensile stress acts in. 

The effect of fiber orientation coefficient on the tensile behavior before cracking is slight, and the difference in the tensile behavior due to the effect is hardly found with ease in experiments with the cementitious composites. 

The major contribution of fibers in fiber reinforced composite is on the tensile behavior after cracking. Post-cracking tensile behavior of the composite can be expressed as a combination of the tensile resistance of the fibers and the matrix. The tensile resistance of a fiber laid across crack surface is calculated considering its inclined angle, shorter embedded length, and the pullout behavior. The resistance by all the fibers is obtained by summing up the pullout resistance of each fiber, with the assumption that all the fibers are not fractured, but pulled out. The resisting force against pullout of a single fiber is presented as a function of its inclination angle (θ), its embedded length ($l_{e}$), and the crack opening displacement of the composite after cracking (δ), which is expressed as $P\left(\theta ,\;l_{e},\;\delta \right)$. Therefore, the fiber bridging stress of the composite at a given crack opening displacement, considering the probability density function of the embedded length and the inclination angle of each fiber being across the cracked plane, can be obtained by Equation (9) [37]:

(9) $$\sigma_{b}\left(\delta \right)=\frac{4V_{f}}{\pi d_{f}^{2}}\int_{0}^{\frac{\pi }{2}}\int_{0}^{\frac{l_{f}}{2}}\;P\left(\theta ,l_{e},\delta \right)\;p\left(l_{e}\right)\;p\left(\theta \right)\cos\theta \;dl_{e}\;d\theta$$

where $p\left(l_{e}\right)$ presents the probability density function for $l_{e}$, and p(θ) is for θ, as mentioned earlier.

An analytical model that could predict the pullout behavior of steel fiber embedded in an ultra-high strength cement-based matrix with an inclination angle was proposed in the study by Lee et al. [38]. The model was adopted for the calculation of the pullout resistance of a single fiber, $P\left(\theta ,l_{e},\delta \right)$. Moreover, it is needed to consider the difference in the bond condition. In their experiment, the pullout of the fiber embedded in a pure matrix without fibers was considered. Whereas the pullout resisting force calculated by Equation (9) is based on the pullout behaviors of the fibers embedded in the fiber-reinforced composites. There is a little discrepancy between the pullout behavior from the pure matrix and the composites [24]. Therefore, a correction factor which can consider the difference was multiplied to the pullout resistance [12].

The tensile resistance by the matrix after cracking should be also considered so as to estimate the post-cracking behavior with the localized crack. Kang and Kim [12] proposed a bilinear tension softening curve for the resistance by the matrix of UHSFRC which can be applied in the prediction of the localized tensile behavior of UHSFRC by combining with fiber bridging resistance. The proposed bilinear tension softening curve is presented by the following equations:

(10) $$\sigma_{mt}=f_{t}\left(1-0.7\frac{w}{w_{c}}\right)\;\mathrm{for}\;0.3f_{t}\leq \sigma_{ct}\leq f_{t}$$

(11) $$\sigma_{mt}=\frac{0.3f_{t}}{w_{c}-w_{1}}\left(w_{c}-w\right)\;\mathrm{for}\;0\leq \sigma_{ct}\leq 0.3f_{t}$$

where w is the crack opening displacement (mm), $f_{t}$ means the tensile strength, and $\sigma_{mt}$ indicates the tensile stress of the matrix at a given crack opening. $w_{1}$ is the crack opening displacement at $\sigma_{mt}=0.3f_{t}$ and it is equal to 0.2 mm. $w_{c}$ is the crack opening displacement at $\sigma_{mt}=0$ and it is equal to 0.5 mm.

## 3. Numerical Simulation Based on the Model

Here, the process of fabricating a rectangular-sectioned UHSFRC beam is considered, and it is intended to predict the variation of the fiber orientation distribution from Equation (1) as the fresh UHSFRC flows and the corresponding variation of fiber bridging behavior, which can be calculated by Equation (9). The rectangular-sectioned beam considered in this study has the dimensions of 100 mm width, 100 mm depth, and 1600 mm length, respectively.

### 3.1. Fiber Orientation Distribution

The fiber orientation distribution according to the flow distance was first estimated with Equation (1). Three-dimensional random distribution of fibers was assumed for the initial condition. For the analytical approach, a fully-developed shear flow between two parallel plates was assumed for the flow field as already explained. The flow volume rate in the entrance, Q was assumed to be 0.001 $m^{3}/s$, which was determined considering the actual flow rate at placement.

**Figure 2:**
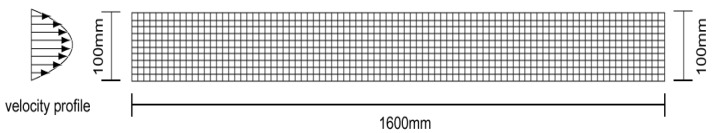
Considered shear flow, number of stream lines, and flow distance lines.

The analyses were carried out from the entrance (left side in Figure 2) to the right end of the beam. The number of streamline is 10 and the flow distance is divided into 100 (Figure 2). After calculating the fiber orientation distribution according to the flow distance for each stream line, the sectional fiber orientation distribution is then calculated by gathering the results of 10 stream lines located at the same distance.

**Figure 3:**
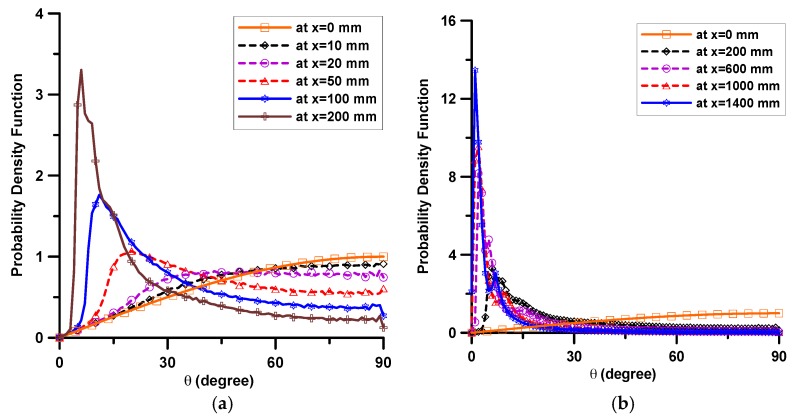
Probability density functions of θ according to the flow distance; (**a**) up to 200 mm flow distance; and (**b**) for overall flow distance.

Figure 3 shows the analytical results: the fiber orientation distribution according to the flow distance, where the fiber orientation indicates the angle between the fiber axis and the shear flow direction. Each section investigated is at a distance of 10, 20, 50, 100, 200, 600, 1000, and 1400 mm, respectively. As shown in Figure 3a, within the initial 200 mm the fiber orientation distribution becomes more concentrated in the range of 0°–20° according to the flow distance from the initial three-dimensional random distribution. Figure 3b demonstrates that, at distances farther than 200 mm, the distributions show similar aspects but the density within 10° increases according to the flow distance. Figure 3 informs that the fibers tend to be arranged in parallel with the flow direction gradually with the increase in the flow distance.

### 3.2. Fiber Bridging Behavior

**Figure 4:**
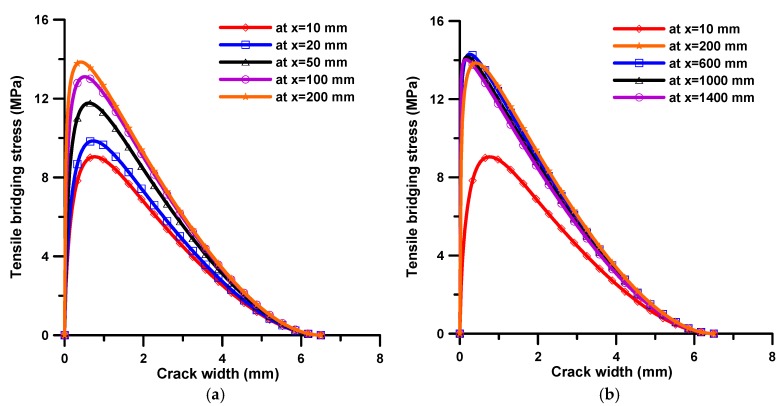
Simulated fiber bridging curves according to the flow distance; (**a**) up to 200 mm flow distance; and (**b**) for overall flow distance.

After estimating the fiber orientation distribution, it was tried to obtain the fiber bridging behavior from the estimated fiber orientation distribution. Figure 4 shows the obtained fiber bridging curves at several flow distances. As can be seen in Figure 4a, the maximum bridging stress improves and the corresponding crack width gets smaller as the flow distance increases up to a 200 mm flow distance. On the other hand, after 200 mm, there is little difference in the bridging behavior, as shown in Figure 4b.

## 4. Experiments

### 4.1. Test Specimen Preparation

The mix proportion of UHSFRC can be seen in Table 1. The water-binder ratio of 0.2 was applied for the mix. Type I Portland cement and silica fume were used for the binder. Fine sand with a maximum grain size 0.5 mm and a density of 2.62 g/cm^3^ was used for fine aggregate. Coarse aggregate was not used. Superplasticizer was included in the mix in order to obtain a satisfactory workability and flowability despite of the low water to binder ratio of UHSFRC. A polycarboxylate-based superplasticizer was used. In addition, the siliceous filler used to enhance the packing density of UHSFRC has mean grain size of about 4 μm. Table 2 provides the material properties of cement, silica fume and filler. Straight steel fibers of $l_{f}$ = 3 mm and $d_{f}$ = 0.2 mm were incorporated by 2 vol % of the composite. 

The workability of the mixture was estimated by the flow table test according to ASTM C 1437. The measured flow of the fresh UHSFRC was 240 mm. The compressive strength and elastic modulus were also measured with three cylindrical specimens with the size of ϕ 100 mm × 200 mm. The compressive strength was 182 to 192 MPa and the elastic modulus was 43.4 to 43.8 GPa. 

**Figure 5:**
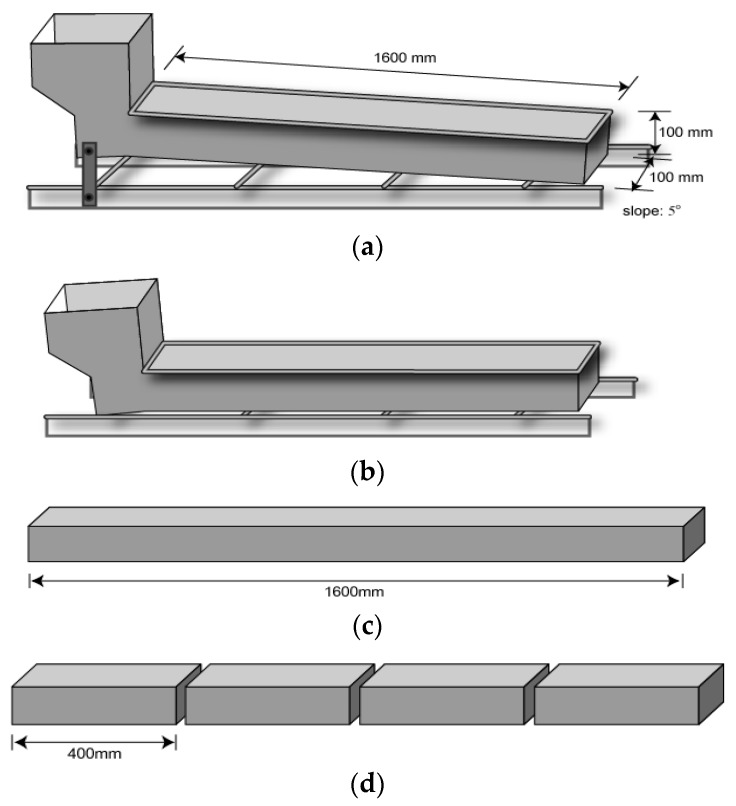
Procedure of specimen preparation; (**a**) a device for manufacturing a long beam with one-side placing; (**b**) on a level surface after placement; (**c**) beam specimen after demolding; and (**d**) divided small specimens after cutting.

For the experimental program, two beams with dimensions of 100 mm × 100 mm × 1600 mm were manufactured. The fresh UHSFRC was devised to be placed at one side of the beam, to flow by itself, and to be filled simultaneously throughout the length, as shown in Figure 5a. After finishing placement, the beam specimens were laid on a level surface (Figure 5b). After demolding and curing, the beam (Figure 5c) was cut and divided into four small specimens with dimensions of 100 mm × 100 mm × 400 mm, as shown in Figure 5d. With the divided specimens, a standard flexural tensile test was performed.

### 4.2. Test Method

**Figure 6:**
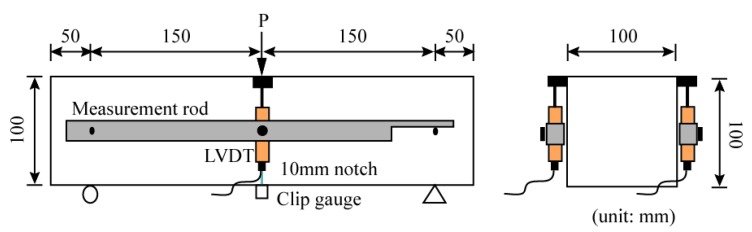
Setup of the flexural tensile test (three-point bending test with a notched beam).

The tensile behavior with different flow distances in the beam structure was evaluated by a flexural tensile test. With four divided small specimens, a three-points bending test was conducted with a 10 mm notch at the midspan. An actuator with 250 kN load capacity was used for the test, and the displacement-controlled loading was adopted. A clip gauge was attached across the notch to measure the crack opening displacement with applied load, as shown in Figure 6. A LVDT (linear variable displacement transducer was also installed at the center of the specimens to measure the central deflection. Figure 6 illustrates the setup employed for the flexural tensile test.

### 4.3. Test Results

**Figure 7:**
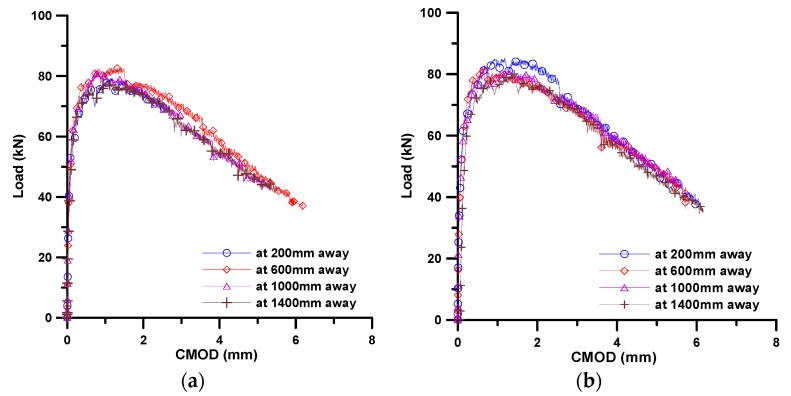
Load-CMOD curves obtained from experiments; (**a**) from beam 1; and (**b**) from beam 2.

Figure 7 presents the flexural test results of four small specimens obtained from each of the two beams. The results were described in terms of the load versus CMOD (crack mouth opening displacement) relation. As can be seen in Figure 7, there is little difference in the flexural behavior with different flow distances. Recalling the results of fiber bridging behavior according to flow distance, shown in Figure 4b, these test results show good correlation with the predicted results of fiber bridging behavior.

## 5. Analysis and Comparison

**Figure 8:**
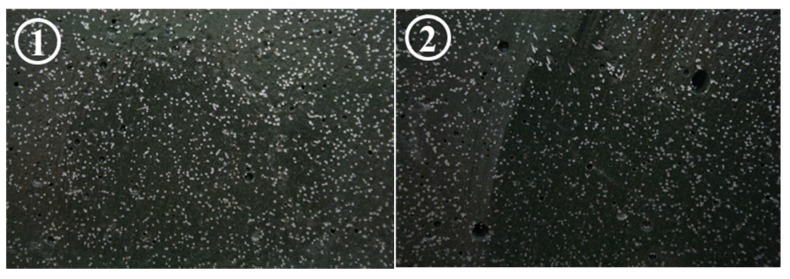

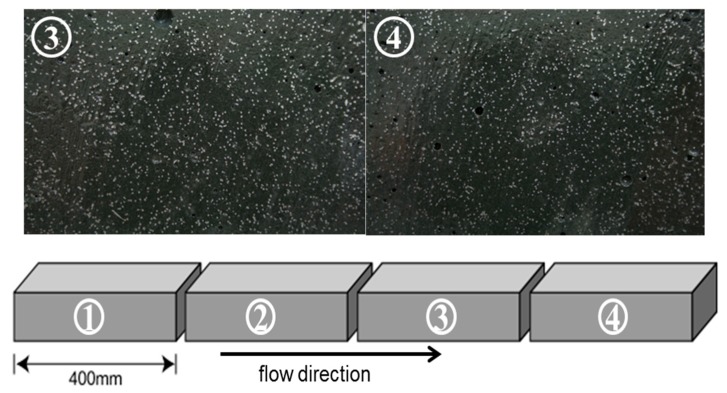
Detected fiber distributions according to the flow distance of UHSFRC.

First, the fiber orientation distribution near the fractured surface in the beam test was investigated in order to examine the relation between the fiber orientation and the flexural behavior. For the purpose, the specimens after finishing the flexural test were cut along a plane near to the fractured plane induced by notch, and sectional images of the cut planes were obtained. Figure 8 presents the fiber distributions observed at the cut sections of beam 1. Similar to the results of the flexural test, it could be found that there was no noticeable difference in the fiber orientation distribution. For quantitative evaluation of the fiber orientation distribution, the number of fibers detected in the cut section was counted using an image analysis technique. It may be better if the fiber orientation distribution could be measured directly from the image analysis, but due to the relatively low resolution of the images (approximately 2000 × 1000 pixels) and rough grinding [29], a reliable result for the fiber orientation distribution could not be obtained. Instead, the number of fibers was calculated from the image because it can be counted with relatively high accuracy even with the low quality of the images. It is well known that the number of fibers is closely related with the fiber orientation distribution [37,39]. Table 3 shows the number of fibers detected in the cut section according to the flow distance in beams 1 and 2. A slight increase in the number of fibers with the increase in the flow distance could be seen, but the difference is less than 10%. Eventually, it demonstrates that the trivial difference in the fiber orientation distribution after 200 mm consequently resulted in the almost consistent flexural behavior regardless of flow distance.

For the flow distance less than 200 mm, even though the fiber orientation distribution was not observed, several relevant studies already revealed that there was a noticeable change in the fiber orientation within several tens of millimeters from the entrance [26,40,41].

**Figure 9:**
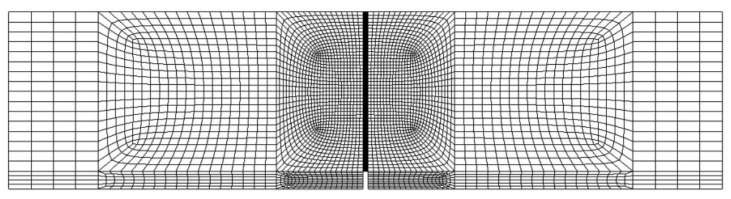
Finite element meshes of the notched beam in FEM analysis.

For the direct comparison of the results of experiment and the prediction, a two-dimensional finite element method (FEM) analysis for the flexural behavior was carried out. In the FEM analysis, in order to simulate the crack propagation from the notch, the fictitious crack model was applied and the interface elements were adopted along the line that the crack propagates. The tensile behavior of the interface elements is linear elastic before cracking, but after cracking it is defined by the combination of the matrix tension softening curve and the fiber bridging curve which is dependent on the flow distance due to the change in the fiber orientation distribution. The other elements were formulated by four-node quadrilateral isoparametric plane stress elements. Figure 9 illustrates the finite element meshes of the notched beam considered in the FEM analysis.

**Figure 10:**
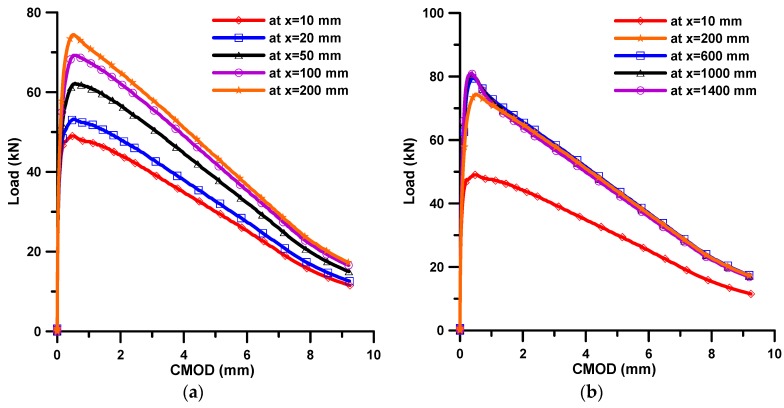
Load-CMOD curves obtained from FEM analysis; (**a**) up to 200 mm flow distance; and (**b**) for overall flow distance.

Figure 10 presents the flexural behaviors obtained from the FEM analysis, which are expressed in load-CMOD curves. As can be expected from the results of fiber bridging behavior (see Figure 4), the maximum load increases as the flow distance increases up to 200 mm flow distance. On the other hand, after 200 mm there is little difference in the bridging behavior, although the peak load at 200 mm flow distance is slightly less than the others. The softening behaviors after roughly 0.5 mm CMOD are almost the same from 200 to 1400 mm.

**Figure 11:**
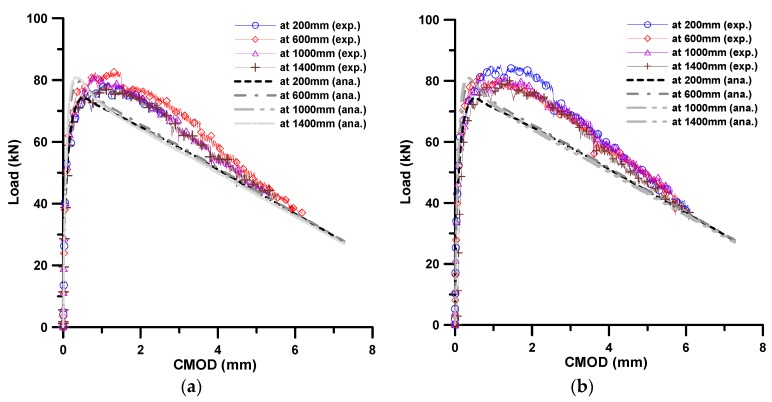
Comparison of flexural behaviors obtained from analysis and experiment; (**a**) from beam 1; and (**b**) from beam 2.

The numerically-estimated results were directly compared with the experimentally-obtained results. In Figure 11, it can be said that the flexural behavior predicted using the analytical fiber bridging curve, which is computed from the estimation of the fiber orientation distribution according to flow, gives reasonable results when compared with the experimental results, especially for the maximum load.

It can be seen that there is a slight difference between the analysis and experiment results with respect to the post-peak behavior, which is due to the difference in the fiber orientation distribution according to the flow. First, it was assumed that there was no interaction among the fibers for simplicity in the simulation of fiber rotational motion, but it is natural that there is some interaction among fibers in practice with 2 vol % fiber volume content. This assumption might cause the difference in the fiber orientation distribution according to the flow distance. Second, in the analysis, the flow was assumed to be an idealized, fully-developed shear flow between two parallel plates with some other assumptions, but the actual flow condition is not like that. A fully-developed flow cannot be accomplished near the entrance, and the flow front actually forms a fountain flow, having two components of velocity in the planar flow. In addition, there exists another boundary condition at the lower surface of the form. Therefore, strictly speaking, the flow is not the same as that between two parallel plates. These differences in the flow aspects might bring about the difference in the fiber orientation distribution between the idealized and actual flow, consequently influencing the flexural behavior. Nevertheless, this analytical approach is still useful for predicting the flow-dependent tensile behavior.

## 6. Conclusions

In this paper, considering the case of fabricating a beam with the method of placing one end and self-flowing to the other end, it was intended to simulate the variation of the fiber orientation distribution according to the flow distance and the variation of the resultant tensile behaviors. Then the validity of the simulation approach was shown by comparing the simulated results with experimental ones. A three-point bending test with a notched beam was adopted for the experiment, and FEM analysis was performed to obtain the simulated results for the bending test considering the flow-dependent tensile behavior of UHSFRC. 

In order to simulate the process of fabricating a rectangular UHSFRC beam, where its dimensions are 100 mm width, 100 mm depth, and 1600 mm length, the flow was assumed to be a fully-developed shear flow between two parallel plates. In this case, the major change in the fiber orientation distribution took place within the initial 200 mm of the flow. The slight variation of the fiber orientation distribution beyond 200 mm was found to have no significant influence on the tensile behavior. Within the limited experimental program of this study, it could be said that the predicted tensile behavior according to the flow distance was tolerably coincident with the experimental results.

## Figures and Tables

**Table 1:** Mix proportion and physical properties of UHSFRC.

Relative Weight Ratios						Steel Fiber (*V_f_*, %)	Physical Properties		
Cement	Water	Silica Fume	Sand	Filler	SP		Flow (mm)	Comp. Strength (MPa)	Elastic Modulus (GPa)
1.00	0.25	0.25	1.10	0.30	0.018	2	240	182 to 192	43.4 to 43.8

SP: superplasticizer and *V_f_*: volume fraction of fiber.

**Table 2:** The properties of cement, silica fume, and filler.

Item		Cement	Silica Fume	Filler
Physical properties	Density (g/m^3^)	3.15	2.10	2.65
	Specific surface area (cm^2^/g)	3413	200,000	N.M.
Chemical composition (%)	SiO_2_	21.01	96.00	99.50
	CaO	61.33	0.38	0.01
	Al_2_O_3_	6.40	0.25	0.38
	Fe_2_O_3_	3.12	0.12	0.04
	MgO	3.02	0.10	0.01

**Table 3:** The number of fibers detected in the cut section according to the flow distance.

Flow Distance (mm)	The Number of Fiber Detected		
	Beam 1	Beam 2	Average
200	3655	3622	3639
600	3455	4032	3744
1000	3675	3829	3752
1400	3959	3944	3951
